# An AluYa5 Insertion in the 3′UTR of *COL4A1* and Cerebral Small Vessel Disease

**DOI:** 10.1001/jamanetworkopen.2024.7034

**Published:** 2024-04-17

**Authors:** Chaker Aloui, Lisa Neumann, Françoise Bergametti, Eric Sartori, Marc Herbreteau, Arnaud Maillard, Thibault Coste, Hélène Morel, Dominique Hervé, Hugues Chabriat, Serge Timsit, Irina Viakhireva, Yves Denoyer, Rémi Allibert, Florence Demurger, Cedric Gollion, Patrick Vermersch, Florence Marchelli, Corinne Blugeon, Sophie Lemoine, Claire Tourtier-Bellosta, Alexis Brouazin, Anne-Louise Leutenegger, Eva Pipiras, Elisabeth Tournier-Lasserve

**Affiliations:** 1 NeuroDiderot, Université Paris Cité, Institut National de la Santé Et de la Recherche Médicale, Unité Mixte de Recherche 1141, Paris, France; 2 Service de Neurologie, Centre Hospitalier Bretagne Sud, Lorient, France; 3 Assistance Publique-Hôpitaux de Paris, Service de Génétique Moléculaire Neurovasculaire, Hôpital Saint-Louis, Paris, France; 4 Assistance Publique-Hôpitaux de Paris, Service de Neurologie, Hôpital Lariboisière, Paris, France; 5 Service de Neurologie Vasculaire, Centre Hospitalier Régional Universitaire de Brest, Brest, France; 6 Université de Rennes, Laboratoire Traitement du Signal et de l'Image, Institut National de la Santé Et de la Recherche Médicale Unité Mixte de Recherche 1099, Rennes, France; 7 Service de Neurologie, Unité Neurovasculaire, Centre Hospitalier Universitaire de Saint Etienne, Saint Etienne, France; 8 Service de Neurologie, Unité Neurovasculaire, Centre Hospitalier Bretagne Atlantique, Vannes, France; 9 Service de Neurologie, Centre Hospitalier Universitaire de Toulouse, Toulouse, France; 10 Univ. Lille, Institut National de la Santé Et de la Recherche Médicale Unité Mixte de Recherche 1172 LilNCog, Centre Hospitalier Universitaire Lille, Fédérations Hospitalo-Universitaire Precise, Lille, France; 11 GenomiqueENS, Institut de Biologie de l’Ecole Normale Supérieur, Département de biologie, École Normale Supérieure, Centre National de la Recherche Scientifique, Institut National de la Santé Et de la Recherche Médicale, Université Paris Sciences et Lettres, Paris, France; 12 Service de neurologie, Centre Hospitalier de Cornouaille, Quimper, France; 13 Assistance Publique-Hôpitaux de Paris, Hôpitaux Jean Verdier et Armand Trousseau, Université Sorbonne Paris Nord, Bobigny, France

## Abstract

**Importance:**

Cerebral small vessel diseases (CSVDs) account for one-fifth of stroke cases. Numerous familial cases remain unresolved after routine screening of known CSVD genes.

**Objective:**

To identify novel genes and mechanisms associated with familial CSVD.

**Design, Setting, and Participants:**

This 2-stage study involved linkage analysis and a case-control study; linkage analysis and whole exome and genome sequencing were used to identify candidate gene variants in 2 large families with CSVD (9 patients with CSVD). Then, a case-control analysis was conducted on 246 unrelated probands, including probands from these 2 families and 244 additional probands. All probands (clinical onset <age 55 years and ≥1 first-degree relative with CSVD) were referred to the French cerebrovascular referral center between 2013 and 2023. The large-scale gnomAD structural variant database and 467 healthy individuals of French ancestry were used as a control group.

**Main Outcomes and Measures:**

A pathogenic AluYa5 insertion was identified within the *COL4A1* 3′UTR in the 2 large families with CSVD. Reverse transcriptase–quantitative polymerase chain reaction (RT-qPCR), Western blot, and long-read RNA sequencing were used to investigate outcomes associated with the insertion using patient fibroblasts. Clinical and magnetic resonance imaging features of probands with variants and available relatives were assessed.

**Results:**

Among 246 probands (141 females [57.3%]; median [IQR] age at referral, 56 [49-64] years), 7 patients of French ancestry carried the insertion. This insertion was absent in 467 healthy French individuals in a control group (odds ratio, ∞; 95% CI, 2.78 to ∞; *P* = 5 × 10^−4^) and 10 847 individuals from the gnomAD structural variant database (odds ratio, ∞; 95% CI, 64.77 to ∞; *P* = 2.42 × 10^−12^). In these 7 patients’ families, 19 family members with CSVD carried the insertion. RT-qPCR and Western blot showed an upregulation of *COL4A1* mRNA (10.6-fold increase; 95% CI, 1.4-fold to 17.1-fold increase) and protein levels (2.8-fold increase; 95% CI, 2.1-fold to 3.5-fold increase) in patient vs control group fibroblasts. Long-read RNA sequencing data showed that the insertion was associated with perturbation in the use of canonical *COL4A1* polyadenylation signals (approximately 87% of isoforms transcribed from the wild type allele vs 5% of isoforms transcribed from the allele with the insertion used the 2 distal canonical polyadenylation signals). The main clinical feature of individuals with CSVD was the recurrence of pontine ischemic lesions starting at an early age (17 of 19 patients [89.5%]).

**Conclusions and relevance:**

This study found a novel mechanism associated with *COL4A1* upregulation and a highly penetrant adult-onset CSVD. These findings suggest that quantitative alterations of the cerebrovascular matrisome are associated with CSVD pathogenesis, with diagnostic and therapeutic implications.

## Key Points

**Question:**

What are the missing gene variants in cerebral small vessel disease (CSVD)?

**Findings:**

This 2-stage study involved linkage analysis among 2 large families, including 9 patients with CSVD, and a case-control study of 2 probands from these families and 244 unrelated probands; linkage analysis and next-generation sequencing identified a pathogenic AluYa5 insertion in *COL4A1* 3′UTR in the families, while the case-control study identified 7 carriers among 246 probands and none in the control group. This insertion was associated with an upregulation of *COL4A1* mRNA and protein through perturbation of *COL4A1* polyadenylation signals usage and with a severe autosomal dominant CSVD.

**Meaning:**

This study found that a novel sequence variation event was associated with *COL4A1* polyadenylation signals usage and severe CSVD through dysregulation of this major vascular matrisome gene, suggesting diagnostic and therapeutic implications.

## Teaser

This case-control study investigated gene variants associated with cerebral small vessel disease.

## Introduction

Cerebral small vessel disease (CSVD) consists of a heterogeneous group of disorders accounting for one-fifth of stroke cases worldwide.^1^ It is also the second leading cause of dementia.^2^ Most CSVDs are sporadic and associated with age and hypertension, but several monogenic forms of the disease have been reported.^3^ The most frequent one is cerebral autosomal dominant arteriopathy with subcortical infarcts and leukoencephalopathy.^4^ In addition to its diagnostic applications, identification of causative variants has proven to be a powerful approach to decipher CSVD mechanisms.^5^ However, in most patients with familial CSVD, the cause remains elusive, precluding genetic counseling and mechanistic studies. Indeed, targeted sequencing of exons and exon-intron boundaries of all known CSVD genes does not identify a causative variant in almost 80% of familial CSVD cases.^6,7^ This strongly suggests that other genes or noncoding variants may be involved in CSVD.

Linkage analysis performed on large multiplex families in combination with Sanger sequencing led to the discovery of genes involved in numerous monogenic diseases.^4,8,9^ The development of whole exome sequencing (WES) and whole genome sequencing (WGS) substantially shortened this approach.

In this study, we combined linkage analysis, WES, and WGS to identify candidate variants in 2 French families with multiple cases of CSVD. We then designed a case-control study to search for statistical association using probands from these 2 families, 244 additional unrelated probands with CSVD, and 467 healthy French individuals in a control group. We also used the large-scale gnomAD structural variant database as a control.

## Methods

All procedures and protocols for this 2-stage study involving linkage analysis and a case-control study complied with the Institut National de la Santé Et de la Recherche Médicale human investigation committee and institutional review board. All patients provided a written informed consent for participation in genetic studies in accordance with French ethical recommendations for genetic study. Consent forms signed by the patients allow the publication of all clinical and MRI information, except photographs and video and audio recordings of patients. This study followed the Strengthening the Reporting of Observational Studies in Epidemiology (STROBE) reporting guideline for observational studies.

**Figure 1.**
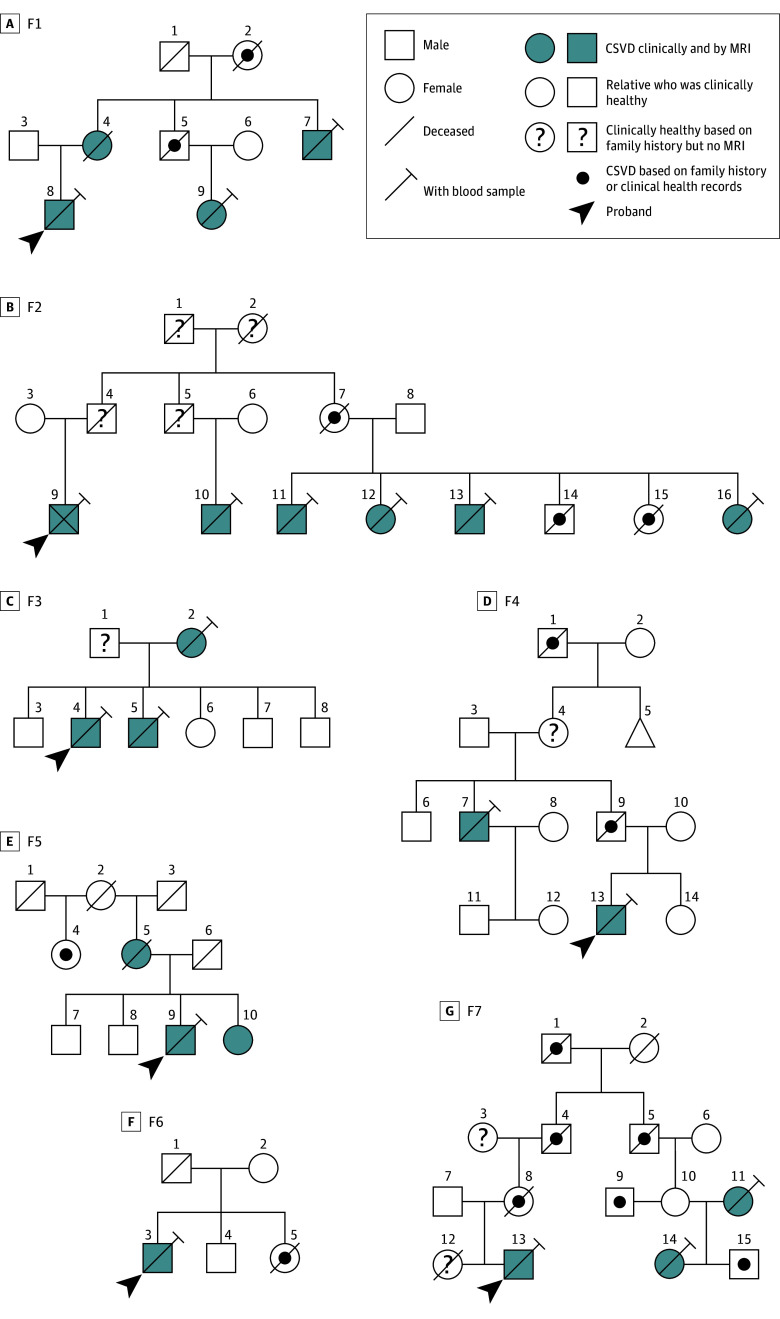
Genealogical Trees of Families F1 to F7 CSVD indicates cerebral small vessel disease; MRI, magnetic resonance imaging.

There were 2 families (F1 and F2), including 3 members in F1 and 7 members in F2 (Figure 1) showing ischemic stroke manifestations and white matter lesions strongly suggestive of a CSVD. They were referred to the French Reference Cerebrovascular Diseases Genetics Lab (Saint-Louis Hospital, Paris). Targeted sequencing of known CSVD genes was performed in F1 and F2 probands. We included 244 additional CSVD probands based on the following criteria: (1) presence of a vascular leukoencephalopathy strongly suggestive of a CSVD; (2) no pathogenic variants detected with our CSVD-targeted gene panel, which includes *NOTCH3*, *HTRA1*, *COL4A1*, *COL4A2*, *TREX1*, *GLA*, *CTSA*, *APP*, and *LAMB1*; (3) age at clinical onset younger than 55 years; and (4) 1 or more first-degree relative with a clinical history of stroke, vascular dementia, or both.

We performed linkage analysis of families F1 and F2 and WES of F1 and F2 family members and 244 additional probands with CSVD as described in the eMethods in Supplement 1. To further investigate structural and regulatory noncoding variants present in possibly linked regions, we performed WGS of the 3 F1 patients (eMethods in Supplement 1).

Copy number variants and structural variations, including mobile element insertions (MEIs), were searched from Illumina single-nucleotide variation (formerly, single-nucleotide polymorphism) arrays and WGS data as described in the eMethods in Supplement 1. Polymerase chain reaction (PCR) and Sanger sequencing (protocols available upon request) were used to confirm the candidate MEI, and PCR was used to genotype this MEI in all 244 probands with CSVD and in 467 healthy French individuals in the control group. In addition, the GnomAD structural variant database version 2.1 was used as a control database for structural variations.^10^

Skin fibroblasts were obtained from patients F1-8 and F2-12 and from an additional proband (F8-16) (eFigure 1 in Supplement 1) referred for targeted sequencing and in whom PCR analysis detected the candidate MEI insertion. Of note, proband F8 was identified after the completion of the initial study and was therefore not included in the case-control study. The search for the insertion using PCR was performed in this proband based on clinical, familial, and magnetic resonance imaging (MRI) similarities with previously identified AluYa5 carriers. In addition, we obtained skin fibroblasts from 4 healthy individuals in a control group (C1-C4) (CBC Biotech), 1 patient with CSVD and a *COL4A1* duplication, and 5 patients with CSVD who did not carry the insertion. These fibroblasts were used for reverse transcriptase–quantitative PCR (RT-qPCR), Western blotting, and long-read RNA sequencing (eMethods in Supplement 1). Details on library preparation, sequencing, and alignment according to the Oxford Nanopore technology are presented in the eMethods in Supplement 1. The aim was to investigate alternative polyadenylation signal (PAS) usage depending on the presence or absence of the MEI in the 3′UTR of *COL4A1* (eMethods in Supplement 1).

### Statistical Analysis

The number of carriers of the candidate AluYa5 insertion was compared between patients with CSVD and those in the control group using the Fisher exact test implemented in R statistical software version 4.2 (R Project for Statistical Computing).^11^ We compared mRNA and protein levels between patients with CSVD and those in the control group using the Mann-Whitney test using Prism software version 9 (GraphPad). Differential use of polyadenylation sites between patients and those in the control group was analyzed with a Mann-Whitney test using R statistical software version 4.2.^11^ A 2-sided *P* value < .05 was considered statistically significant.

## Results

### Baseline Characteristics of Study Participants

The 2 large families used for linkage analysis were of French ancestry and included 9 patients with CSVD who had blood samples (3 females [33.3%]; median [IQR] age, 50 [42-59] years). Their genealogical trees are presented in Figure 1. Including 2 probands from the 2 families with CSVD and 244 additional probands, a total of 246 probands (141 females [57.3%]; median [IQR] age at referral, 56 [49-64] years) were used for the case-control study; among them, 227 individuals (92.2%) were of European ancestry. Proband age at clinical onset was younger than 55 years, and probands had at least 1first-degree relative with CSVD.

### Clinical and Neuroimaging Features of Patients in Families F1 and F2

**Figure 2.**
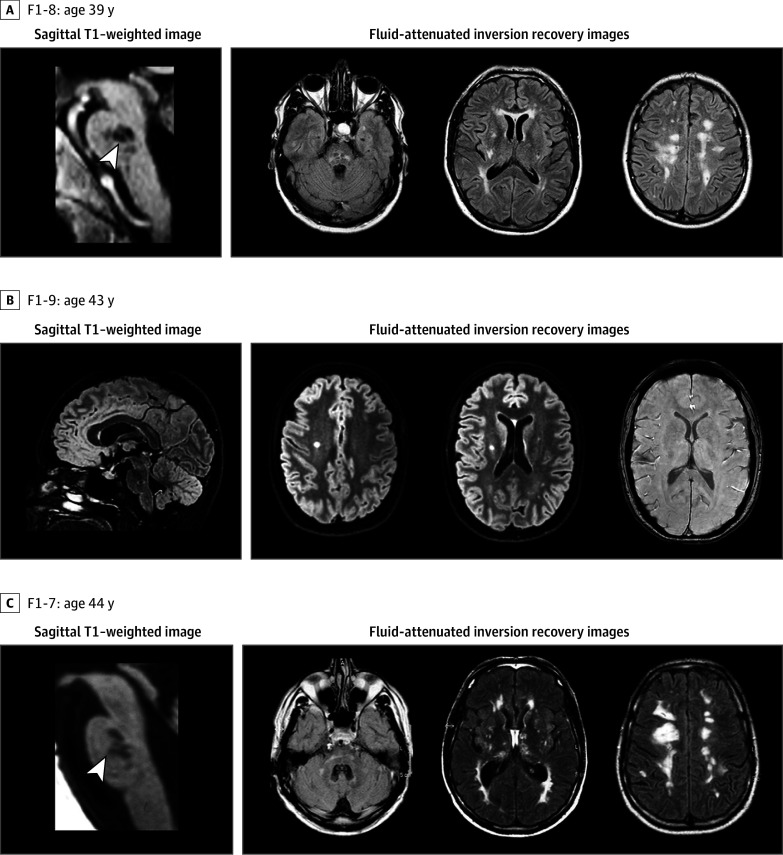
Magnetic Resonance Imaging Data of Patients From Family F1 The first column presents sagittal T1–weighted images. Fluid-attenuated inversion recovery images are presented in columns 2 to 4. All patients present pontine infarcts and a vascular leukoencephalopathy associated with hemispheric lacunes.

**Figure 3.**
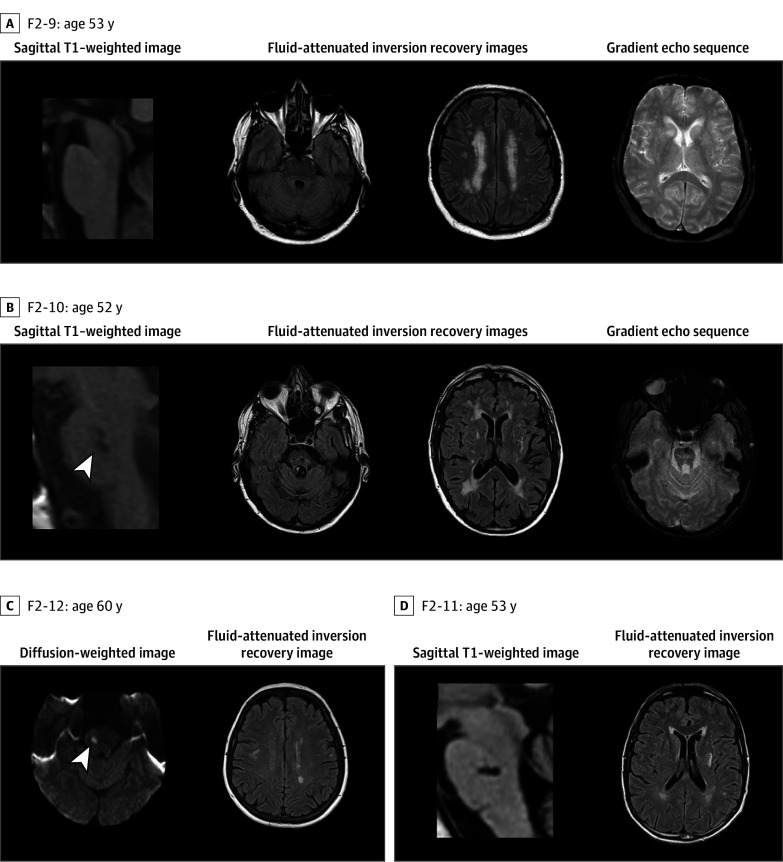
Magnetic Resonance Imaging Data of Patients From Family F2 The first column presents sagittal T1–weighted images except for C (patient F2-12), which presents diffusion-weighted images. Fluid-attenuated inversion recovery images are presented in columns 2 to 4, except for A (patient F2-9) and B (patient F2-10) in column 4, which present gradient echo sequences, and D (patient F2-11) in column 3, which presents sagittal T1 images. All patients except patient F2-9 (A) present pontine infarcts and vascular leukoencephalopathy associated with hemispheric lacunes.

Genealogical trees and clinical and MRI features of F1 and F2 family members are presented in Figure 1, Figure 2, and Figure 3 and eTables 1 and 2 in Supplement 1. Probands F1-8 and F2-9 were referred for targeted CSVD gene screening and remained negative. We investigated 2 additional members of family F1 and 5 additional members of family F2, obtaining clinical and MRI data. In total, blood samples were taken from 9 individuals with CSVD in these 2 families.

#### Family F1

Patient F1-8 (proband) was a male aged 45 years without any vascular risk factor. At age 39 years, he experienced sudden dizziness, paresthesia of the left arm, left central facial palsy, and dysarthria related to a pontine ischemic lesion. At ages 41 and 43 years, he had sudden transient diplopia without any incident lesion on MRI. In the following years, he reported mild cognitive disturbances.

His female first cousin aged 45 years (F1-9) had a well-controlled hypertension under monotherapy but no other vascular risk factor. At age 35 years, she presented a sudden episode of hemisensory loss related to a pontine ischemic lesion. At age 41 years, she had a similar episode. MRI showed an extension of the previous pontine ischemic lesion and an additional punctiform white matter hyperintensity in the centrum semioval.

Their uncle (F1-7) was an active smoker and already experienced myocardial infarction. At age 42 years, he twice presented a sudden episode of loss of balance and dysphonia associated with headache. MRI showed a pontine ischemic lesion. In the 2 following years, he developed a progressive cognitive deterioration and dementia, without any stroke event. He was treated for a primary cerebral vasculitis with cyclophosphamide and corticosteroid for 2 years.

MRI data for these 3 patients are presented in Figure 2. We detected 1 or several lacunes in the pons, subcortical hemispheric area, or both in all these patients. Hemispheric white matter hyperintensities were present in all patients, in the centrum semioval, subcortical, and periventricular areas. Anterior temporal lobes, thalami, and external capsules were involved in the 2 most severe cases. Gradient echo images, available in only F1-9, did not detect any microbleed.

In addition to these 3 patients, 3 additional patients (F1-4, F1-5, and F1-2) were assessed using clinical health records. The proband’s mother (F1-4) presented at ages 49 and 51 years with 2 episodes of sudden loss of balance and vertical diplopia and developed cognitive impairment. F1-5, another uncle of the proband, presented with a stepwise motor and cognitive deterioration up to a motor disability with dementia. He was treated for a long time as having progressive multiple sclerosis. F1-2, the grandmother of the proband, had a history of early severe cognitive impairment. F1-4, F1-5, and F1-2 died prematurely at ages 53, 62, and 50 years, respectively.

#### Family F2

Proband F2-9, a male aged 55 years, had several vascular risk factors, including hypertension (well-controlled by treatment), dyslipidemia, active smoking, and alcoholism. He complained with chronic headache and developed a stepwise cognitive decline from age 50 years. Cognitive testing showed alteration of performance, possibly increased by an anxiety disorder, in tests evaluating attention, processing speed, and verbal episodic memory. No sudden focal symptom was ever observed. His first MRI (at age 53 years) showed a recent ischemic lesion in the right lenticular nucleus. In 2022, he experienced diplopia (sixth nerve palsy) and gait disturbance.

Patient F2-10 experienced a transient episode of diplopia at age 42 years and a partial third nerve palsy at age 45 years, both related to pontine ischemic lesions. At ages 46 and 49 years, he presented a sudden hemiparesis related to incident ischemic lesions in the centrum semioval and internal capsule. He was treated for 4 years as having progressive multiple sclerosis. At age 56 years, he died because of a severe deep intracerebral hemorrhage.

There were 4 other first cousins of F2-9 who were investigated and underwent MRI and blood sampling. Patient F2-11 presented at age 54 years with a sudden diplopia, ptosis, and loss of balance related to a recent pontine ischemic lesion. At age 59 years, patient F2-12 experienced a sudden episode of transient third nerve palsy with loss of balance. At age 59 years, patient F2-13 presented a pontine infarct. Patient F2-16 experienced an episode of numbness of the left hemibody with sudden gait disturbance at age 60 years.

Of these 6 patients, 5 individuals presented lacunes located in the pons, including 3 patients with multiple pontine ischemic lesions. The sixth patient (F2-12) had no pontine lesion but experienced symptoms evocative of vertebrobasilar territory transient ischemic attack. There were 4 patients with lacunes located in the subcortical hemispheric area. Hemispheric white matter hyperintensities were observed in all 6 patients, although with variable magnitude. No abnormalities were observed in the anterior temporal lobes. Gradient echo images, available in all patients, showed microbleeds in 1 patient (F2-10) in the deep supratentorial gray matter, deep cerebellum, and brainstem.

Clinical health records were obtained for 2 additional patients. Patient F2-14 had a vascular leukoencephalopathy and died prematurely at age 55 years. Patient F2-15 died prematurely at 56 years of a cerebral hemorrhage.

### Linkage Analysis and WES Data

We identified 35 and 22 possibly linked regions for family F1 and F2, respectively (eFigures 2-3 in Supplement 1). Our WES filtering strategy identified 1 variant fulfilling our pathogenicity criteria (eMethods in Supplement 1). This variant, detected in F2, was located in a zinc transporter gene expressed in the mammary gland and was therefore not further considered. Linkage analysis revealed that the families shared a 4.8-Mb possibly-linked region on 13q33-34, a locus containing *COL4A1* and *COL4A2*. In cDNA sequencing of *COL4A1* and *COL4A2*, both alleles were expressed and normal in patients F1-8 and F2-12. Altogether, these data suggested that a noncoding sequence variation may have been located at the *COL4A1/COL4A2* locus.

### WGS Data Analysis

WGS did not detect any pathogenic variant in the *COL4A1/COL4A2* promoter region. Structural variant search using Delly, Lumpy, and Manta was negative within the *COL4A1/COL4A2* region and in other linked regions. Careful visualization of reads aligned within the *COL4A1/COL4A2* locus revealed the presence of an insertion, which was exclusively supported by soft-clipped reads, in the 3′UTR of *COL4A1* (eFigure 4 in Supplement 1). The insertion at position chr13:110149065 (hg38) best matched to the consensus sequence of AluYa5, with the presence of 2 single substitutions (C110T and A189G). The insertion was validated by PCR, Sanger sequencing, and long-read RNA sequencing (eFigures 5-6 in Supplement 1).

### AluYa5 Insertion in 3′UTR of *COL4A1* and CSVD

In addition to patients from F1 and F2, 5 additional probands with CSVD who were unrelated also had the insertion. Results were confirmed by PCR and Sanger sequencing. We conducted PCR genotyping on 467 healthy French individuals in a control group to evaluate the prevalence of this genetic variant within the healthy French population to exclude that it may be a benign polymorphism in the French population. All individuals in this control group tested negative for the variant (odds ratio, ∞; 95% CI, 2.78 to ∞; *P* = 5 × 10^−4^), suggesting its rarity or absence in the general French population. Furthermore, we investigated the presence of our insertion in the gnomAD structural variant database version 2.1, in which the same software (ie, MELT) was used to identify Alu insertions. The insertion was not found in 10 847 well-covered genomes in the 3′UTR of *COL4A1* (odds ratio, ∞; 95% CI, 64.77 to ∞; *P* = 2.42 × 10^−12^).

### Upregulation of *COL4A1* mRNA and Protein in Carriers of AluYa5

To study functional outcomes associated with the AluYa5 insertion, we compared *COL4A1* mRNA and protein levels in fibroblasts from patients F1-8 and F2-12, healthy individuals C1 to C4 in a control group, a *COL4A1/2* duplicated patient, and a CSVD patient without the insertion as a control. RT-qPCR showed a 10.6-fold (95% CI, 1.4-fold to 17.1-fold) increase of *COL4A1* mRNA levels in patients F1-8 and F2-12 vs individuals in the control group (eFigure 7 in Supplement 1).

**Figure 4.**
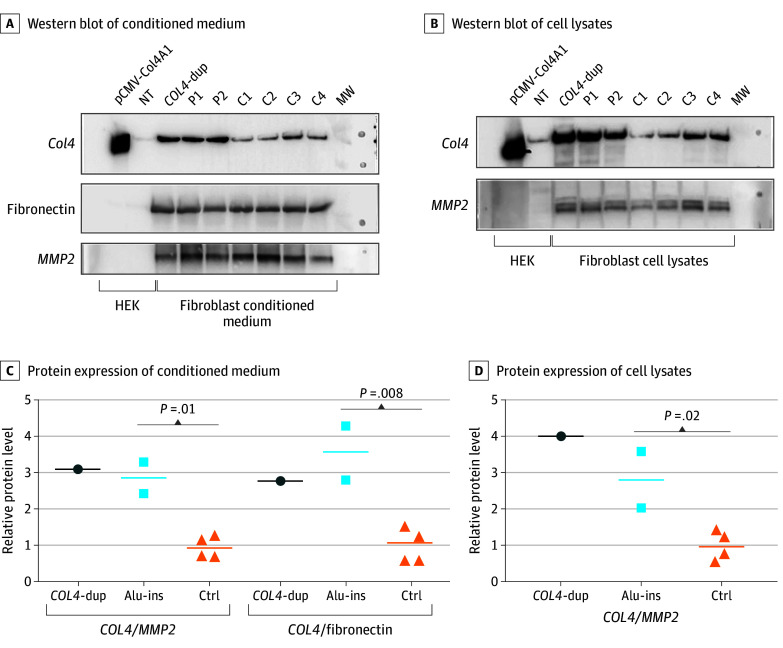
Western Blot Analysis of Wild-Type and Variant *COL4A1* Expressed in Transfected Cells and Endogenous Fibroblasts A-B, A Western blot of conditioned medium and cell lysates from HEK293T cells transfected with wild type and variant *COL4A1* and human skin fibroblasts from 4 healthy individuals in the control group (C1-C4), 2 patients carrying the AluYa5 insertion (P1-P2), and 1 patient duplicated at the *COL4A1/2* locus. A, In the top panel, blot n°1 was probed with an anticollagen IV antibody, and in the middle and bottom panels, blot n°2 was probed with antifibronectin and anti-*MMP2* antibodies used as loading controls (see antibody references in the eMethods in Supplement 1). C-D, Visualizations of relative protein expression levels are presented from A and B, respectively. Circles indicate *COL4*-dup, the patient duplicated at the* COL4A1/2* locus; HEK, HEK293T cells; horizontal lines, means; MW, molecular weight; NT, nontransfected plasmid; pCMV-Col4a1, plasmid-expressing *COL4A1*; squares, patients with AluYa5 insertion; triangles, control group.

Protein levels of *COL4A1* were also significantly higher in fibroblast lysates (2.8-fold; 95% CI, 2.1-fold to 3.5-fold increase) and conditioned media (2.9-fold; 95% CI, 2.4-fold to 3.3-fold increase) from patients with the insertion compared with 4 healthy individuals in the control group. These levels were comparable with those detected in the duplicated patient who served as a control (Figure 4).

### AluYa5 Insertion and PAS Usage

**Figure 5.**
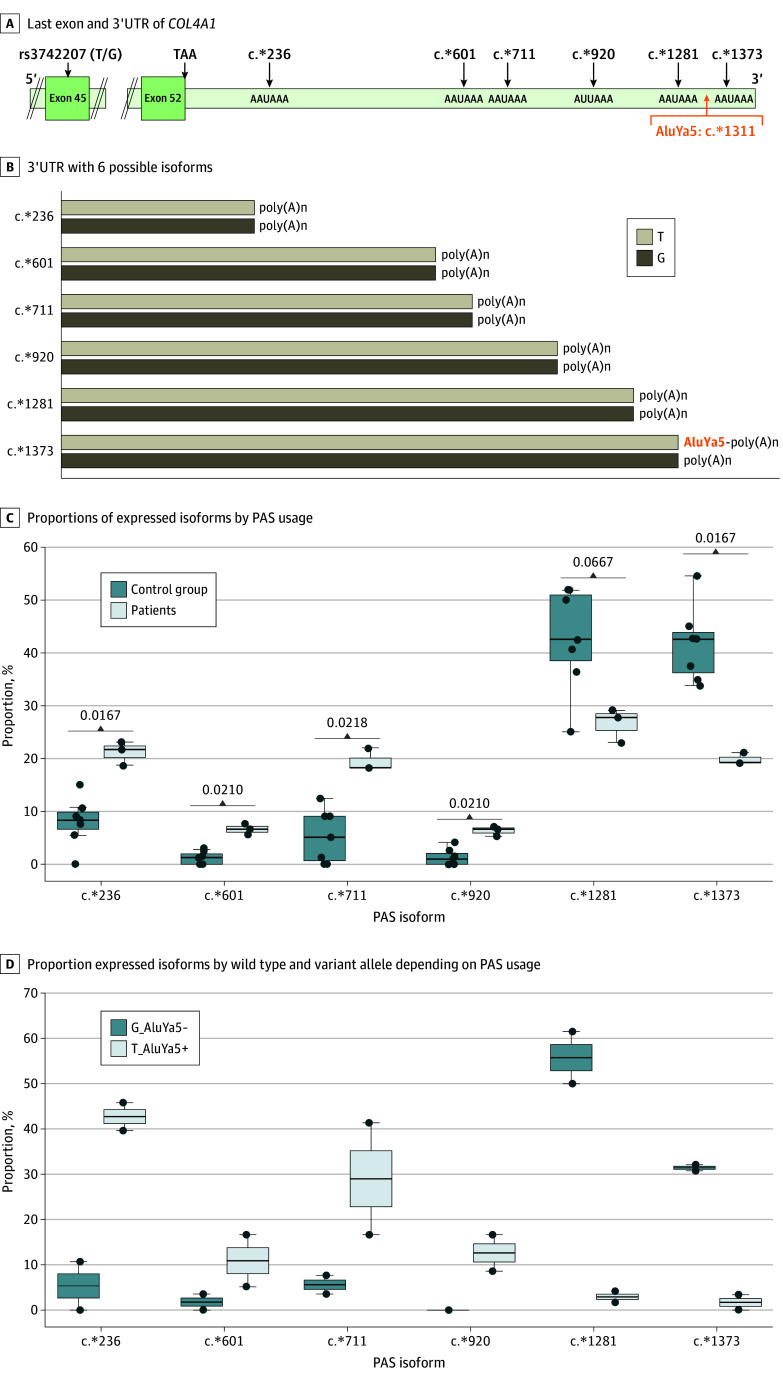
AluYa5 Insertion and Polyadenylation Signal (PAS) Usage A, The diagram shows the last exon and 3′UTR of *COL4A1* with the 6 PAS annotated as c.*distance to the stop codon. B, The diagram of the 3′UTR shows the 6 possible isoforms depending on PAS usage. The 6 isoforms differ only by the length of their 3′UTR. Cleavage and polyadenylation start approximately 15 to 30 bp downstream of the PAS. C, Proportions of expressed isoforms depending on PAS usage are shown in 3 patients carrying the insertion and 7 individuals in the control group. D, Proportions of each of expressed isoform by the wild type and variant allele depending on PAS usage in patients F2-12 and F8-16 are presented. T_AluYa5+ represents isoforms transcribed from the allele that carries the AluYa5 insertion. G_AluYa5− represents isoforms transcribed from the wild type allele.

Based on the location of this insertion within the 3′UTR region, we raised the hypothesis that it may have been associated with disruption of *COL4A1* PAS usage.^12,13^ The National Center for Biotechnology Information (NCBI)^14^ lists 2 PASs for *COL4A1*; the major one is located at position c.6421 (designated in Figure 5 as c.*1281 based on its distance to the stop codon) and the second one at position c.6513 (c.*1373). PAS databases (eg, PolyA_DB3^15^) list more than 10 PASs for *COL4A1*. We used long-read RNA sequencing of 3 patients with the insertion (F1-8, F2-12, and F8-16) and 7 individuals in a control group (4 healthy individuals and 3 patients with CSVD without the insertion) to investigate the usage of PASs in these individuals, finding that 6 isoforms of *COL4A1* could be identified depending on PAS usage (eFigure 8 in Supplement 1). They differed in their 3′UTR size. Among 7 individuals in the control group, mainly 2 isoforms using the 2 distal canonical PASs listed in NCBI were expressed, with approximately 42% use of the c.*1281 PAS and approximately 43% use of the c.*1373 PAS (Figure 5). In contrast, 3 patients with the insertion expressed equally 4 isoforms, with approximately 23% usage of each of 2 distal PASs (c.*1281 and c.*1373) but also approximately 20% usage of c.*236 and c.*711 proximal sites. We detected 2 other isoforms (c.*610 and c.*920) at an approximately 7% level in the patients that were not expressed or were expressed at a very low level in individuals in the control group (Figure 5).

In addition, we took advantage of a heterozygous polymorphism present in *COL4A1* exon 45 (rs3742207-T/G) in patients F2-12 and F8-16 to quantify transcripts encoded by the wild type allele and inserted allele using long-read RNA sequencing data. The insertion occurred on the same haplotype as the T allele (T_AluYa5 haplotype). Proportions of isoforms produced by the T_AluYa5 allele were as follows: approximately 43% c.*236, approximately 10% c.*601, approximately 29% c.*711, and approximately 13% c.*920; approximately 5% of transcripts used the 2 distal signals c.*1281 and c.*1373. Proportions of isoforms produced by the wild type allele (G allele/no-AluYa5 insertion) were similar to those observed in the control group; approximately 87% of isoforms used the 2 canonical distal PASs. The remaining approximately 13% of isoforms used the 4 other PASs (Figure 5). Altogether, these RNA sequencing and expression data suggest that the Alu insertion was associated with changes in usage of PAS and an upregulation of *COL4A1* mRNA and protein levels.

### Clinical and Neuroimaging Features of F3-F7 Patients

Genealogical trees and clinical and MRI features of F3 to F7 probands and relatives are presented in Figure 1 and eFigure 9 and eTables 3 to 4 in Supplement 1. Familial history was suggestive of an autosomal dominant inheritance in all families (Figure 1). Clinical and MRI features were similar to those observed in families F1 and F2, with pontine involvement and hemispheric multiple ischemic lesions associated with hemispheric white matter hypersignals in most patients (9 of 10 patients [90.0%]). Recurrent pontine and subcortical ischemic lesions started at a mean (SD) age of 50 (9) years (eTables 1-4 in Supplement 1) among 17 of 19 patients (89.5%). A younger age at onset (<50 years) was observed in 9 of 19 patients. However, microbleeds were more frequent in patients from families F3 to F7. Intracerebral hemorrhage was observed in patients F2-10, F2-15, F5-5, and F5-10.

## Discussion

In this 2-stage study with linkage analysis and a case-control study, we identified an Alu insertion in the 3′UTR noncoding region of *COL4A1* in 7 families with a disabling autosomal dominant CSVD. This insertion was associated with a strong upregulation of *COL4A1* through a change in PAS usage.

Several results support the association of this AluYa5 insertion with CSVD. First, the insertion was absent from the gnomAD structural variant large-scale control database. Second, this insertion was co-segregating with the affected phenotype in all 7 families. Third, this insertion was associated with a strong upregulation of *COL4A1* mRNA and protein levels, reminiscent of pontine autosomal dominant microangiopathy arteriopathy with leukoencephalopathy (PADMAL), another severe CSVD caused by a sequence variation in a *COL4A1* microRNA binding site (miR29).^9,16,17^

Clinical features of patients from these families were characterized by the recurrence of pontine and subcortical ischemic lesions starting at a mean (SD) age of 50 (9) years, and 9 of 19 patients investigated so far had an age of onset younger than 50 years. The high frequency of pontine infarcts has also been observed in PADMAL.^9,16,17^ However, microbleeds (and to a lower degree, intracerebral hemorrhage, as observed in patients F2-10, F2-15, F5-5, and F5-10) may be more frequent in this condition associated with the AluYa5 insertion. Interestingly, *COL4A1* duplications have also been associated with a similar phenotype.^18,19^ Altogether, these data strongly suggest that *COL4A1* noncoding sequence variations should be searched in unresolved familial CSVD cases with pontine infarcts and leukoencephalopathy. This search should combine copy number analysis and 3′UTR screening. When negative, an upregulation of *COL4A1* in patient fibroblasts should be assessed using RT-qPCR and Western-blotting. In case of an upregulation, full sequencing of all noncoding regions should be performed given that other mechanisms may lead to gene upregulation.^20,21^

The Alu insertion identified in these 7 families was associated with strong upregulation of *COL4A1* mRNA and associated with a change in PAS usage in the 3′UTR of this gene. RNA processing is an essential step in gene regulation. It involves an RNA endonucleolytic cleavage followed by the addition of a poly(A) tail, which allows the translocation of the nascent mRNA from the nucleus to the cytoplasm and the regulation of translation efficiency and RNA degradation.^22,23^ This tightly regulated process fine-tunes gene expression in a cell type and cellular state–dependent manner.^12,24,25^ Various loss and gain of function alterations have been reported to be associated with dysregulation of alternative polyadenylation and disease, including endocrine, oncological, immunological, and neurological diseases.^22,23,25,26,27^ As an example, sequence variants in the proximal canonical PAS of the *NAA10* gene lead to an increase in usage of a distal PASs and a 50% decrease in mRNA expression.^28^ In contrast, a variant creating a canonical proximal PAS in *IRF5* shifts polyadenylation from the distal canonical PAS, leading to increased expression of *IRF5*.^29^ The AluYa5 insertion observed in our patients is located downstream of the major canonical distal PAS of *COL4A1*, which is associated with an almost complete abolition of this canonical distal PAS usage and the use of 2 proximal PASs, leading to the expression of shorter 3′UTR isoforms of *COL4A1* and upregulation of *COL4A1* mRNA. Suggesting how this preferential usage of these proximal PAS may be associated with *COL4A1* upregulation would be speculative at this point. However, several lines of evidence obtained in other diseases and cellular models strongly suggest that this upregulation may be an outcome associated with the deletion of regulatory regions in shorter 3′UTRs.^12,21,23,27^

### Limitations

This study has several limitations. The number of patients in these 7 families is still limited, and the description of all phenotypic features of this condition will need the recruitment of additional families. A search for a founding effect will also be needed. This study is monocentric and focused on a singular population. Analysis of other populations is warranted. Nonetheless, this study demonstrates the power of combining linkage analysis of families with several members who have a rare CSVD with WES and WGS from a large series of unrelated probands.

## Conclusions

In this 2-stage study involving linkage analysis of 2 large families and a case-control study of 246 unrelated probands, we found an association between an AluYa5 insertion located in a *COL4A1* 3’UTR and CSVD. We also uncovered a novel mechanism associated with *COL4A1* expression. This discovery suggests the possibility that other, yet-unidentified noncoding anomalies contributing to *COL4A1* upregulation may be responsible for additional familial cases that remain unresolved.

## Notes

Supplement 1. **eFigure 1.** Genealogical Tree of Family F8 **eFigure 2.** Genome-Wide Linkage Analysis of Family F1 **eFigure 3.** Genome-Wide Linkage Analysis of Family F2 **eFigure 4.** Identification of an Insertion Within the 3’UTR of COL4A1 Using Integrative Genomics Viewer **eFigure 5.** Identification of Insertion as Being an AluYa5 Insertion **eFigure 6.** Long Reads From RNA Sequencing Showing AluYa5 Insertion in 3’UTR of COL4A1 **eFigure 7.** Reverse Transcriptase–Quantitative Polymerase Chain Reaction Analysis of Wild-Type and Variant COL4A1 Expressed in Endogenous Fibroblasts **eFigure 8.** Polyadenylation Signal Usage Based on Long-Read RNA Sequencing Data Analysis With Integrative Genomics Viewer **eFigure 9.** Magnetic Resonance Imaging Data of Probands From Families F3-F7 **eTable 1.** Clinical Features of Family F1 Patients **eTable 2.** Clinical Features of Family F2 Patients **eTable 3.** Clinical Features of Family F3 to F7 Probands **eTable 4.** Clinical Features of Family F3 to F7 Relatives With Cerebral Small Vessel Disease **eMethods.** Detailed Methods
eReferences.

Supplement 2.
Data Sharing Statement
