# Supplementary material for: An AluYa5 Insertion in the 3′UTR of COL4A1 and Cerebral Small Vessel Disease
Source: JAMA Netw Open. 2024 Apr 17;7(4):e247034. doi: 10.1001/jamanetworkopen.2024.7034 (PMC11024774; doi:10.1001/jamanetworkopen.2024.7034)
Supplement: Supplement 1. — eFigure 1. Genealogical Tree of Family F8 eFigure 2. Genome-Wide Linkage Analysis of Family F1 eFigure 3. Genome-Wide Linkage Analysis of Family F2 eFigure 4. Identification of an Insertion Within the 3’UTR of COL4A1 Using Integrative Genomics Viewer eFigure 5. Identification of Insertion as Being an AluYa5 Insertion eFigure 6. Long Reads From RNA Sequencing Showing AluYa5 Insertion in 3’UTR of COL4A1 eFigure 7. Reverse Transcriptase–Quantitative Polymerase Chain Reaction Analysis of Wild-Type and Variant COL4A1 Expressed in Endogenous Fibroblasts eFigure 8. Polyadenylation Signal Usage Based on Long-Read RNA Sequencing Data Analysis With Integrative Genomics Viewer eFigure 9. Magnetic Resonance Imaging Data of Probands From Families F3-F7 eTable 1. Clinical Features of Family F1 Patients eTable 2. Clinical Features of Family F2 Patients eTable 3. Clinical Features of Family F3 to F7 Probands eTable 4. Clinical Features of Family F3 to F7 Relatives With Cerebral Small Vessel Disease eMethods. Detailed Methods eReferences. [file jamanetwopen-e247034-s001.pdf]

Supplemental Online Content

Aloui C, Neumann L, Bergametti F, et al. An AluYa5 insertion in the 3′UTR of *COL4A1* and cerebral small vessel disease. *JAMA Netw Open.* 2024;7(4):e247034.
doi:10.1001/jamanetworkopen.2024.7034

eFigure 1. Genealogical Tree of Family F8

eFigure 2. Genome-Wide Linkage Analysis of Family F1

eFigure 3. Genome-Wide Linkage Analysis of Family F2

eFigure 4. Identification of an Insertion Within the 3’UTR of COL4A1 Using Integrative Genomics Viewer

eFigure 5. Identification of Insertion as Being an AluYa5 Insertion

eFigure 6. Long Reads From RNA Sequencing Showing AluYa5 Insertion in 3’UTR of COL4A1

eFigure 7. Reverse Transcriptase–Quantitative Polymerase Chain Reaction Analysis of Wild-Type and Variant COL4A1 Expressed in Endogenous Fibroblasts

eFigure 8. Polyadenylation Signal Usage Based on Long-Read RNA Sequencing Data Analysis With Integrative Genomics Viewer

eFigure 9. Magnetic Resonance Imaging Data of Probands From Families F3-F7

eTable 1. Clinical Features of Family F1 Patients

eTable 2. Clinical Features of Family F2 Patients

eTable 3. Clinical Features of Family F3 to F7 Probands

eTable 4. Clinical Features of Family F3 to F7 Relatives With Cerebral Small Vessel Disease

eMethods. Detailed Methods

eReferences.

This supplemental material has been provided by the authors to give readers additional information about their work.

eFigure 1: Genealogical tree of family F8. F8-16 proband was referred for gene targeted sequencing, after insertion identification in the 7 families reported in the results section. PCR analysis detected the insertion in this patient (F8-16) and her relative F8-12. Square = male; circle = female; diagonal black line = deceased individual; black filled symbol = clinically and MRI-proven affected individual; empty symbol = clinically healthy relative with a normal MRI; empty symbol with a question mark = clinically healthy individuals based on family history but no MRI performed; black dot = affected individual based on family history and/or clinical charts; arrow symbol = probands; syringe symbol = blood-sampled individual; MRI = magnetic resonance imaging.

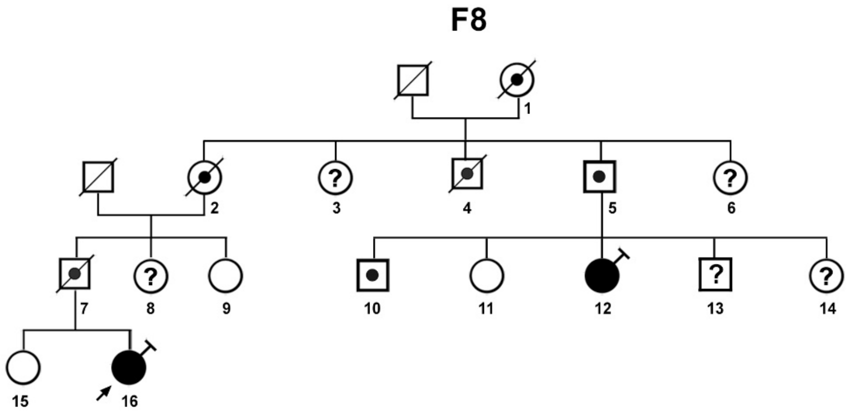

© 2024 Aloui C et al. *JAMA Network Open.*

eFigure 2: Genome-wide linkage analysis of family F1. Multipoint linkage analysis was performed by Merlin software under an autosomal dominant model. The maximum theoretical LOD score for this family was 0.903. X-axis: chromosomal position in centimorgans (cM); Y-axis: LOD score.

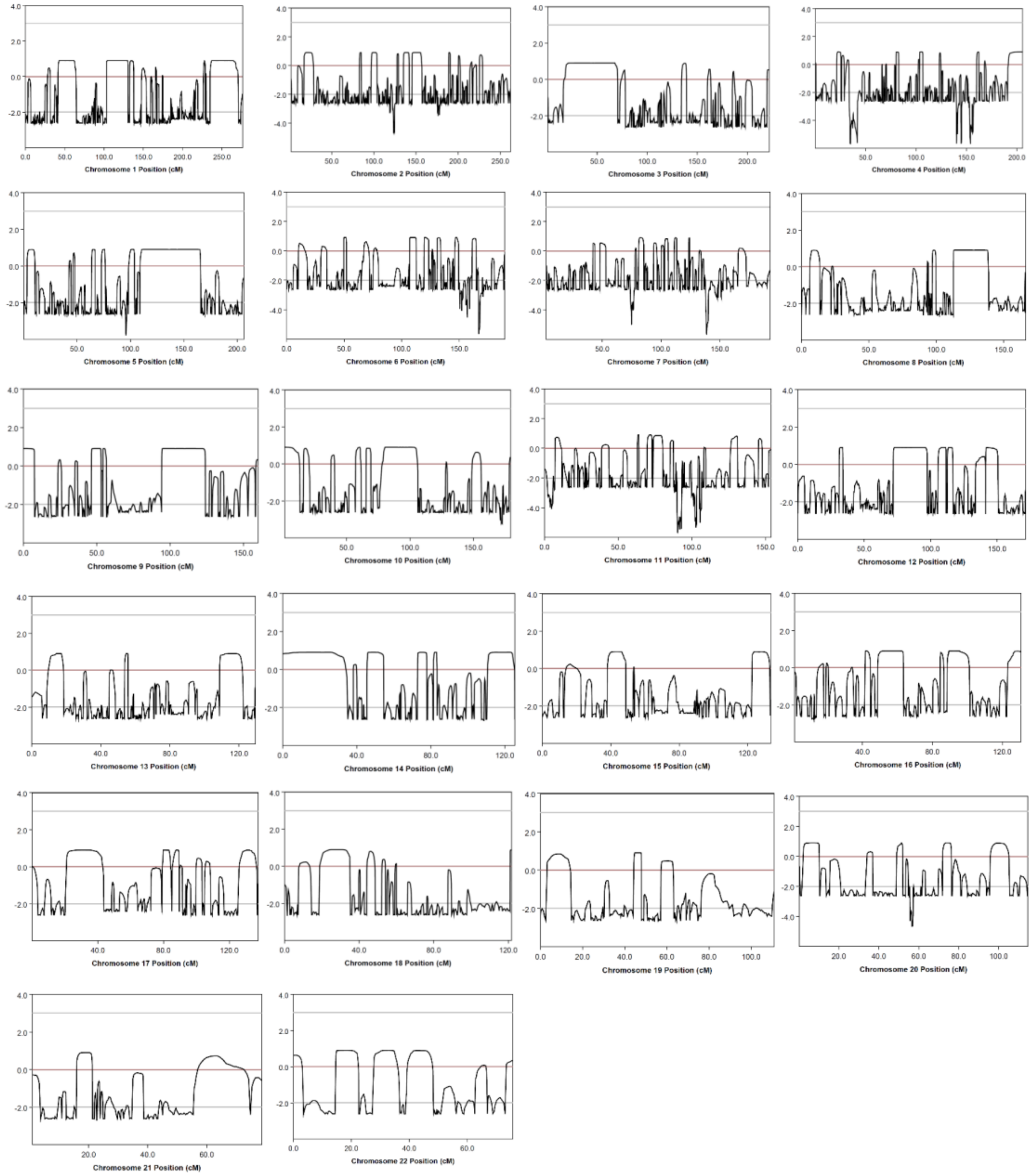

© 2024 Aloui C et al. *JAMA Network Open.*

eFigure 3: Genome-wide linkage analysis of family F2. Multipoint linkage analysis was performed by Merlin software under an autosomal dominant model. The maximum theoretical LOD score for this family was 1.805. X-axis: chromosomal position in centimorgans (cM); Y-axis: LOD score.

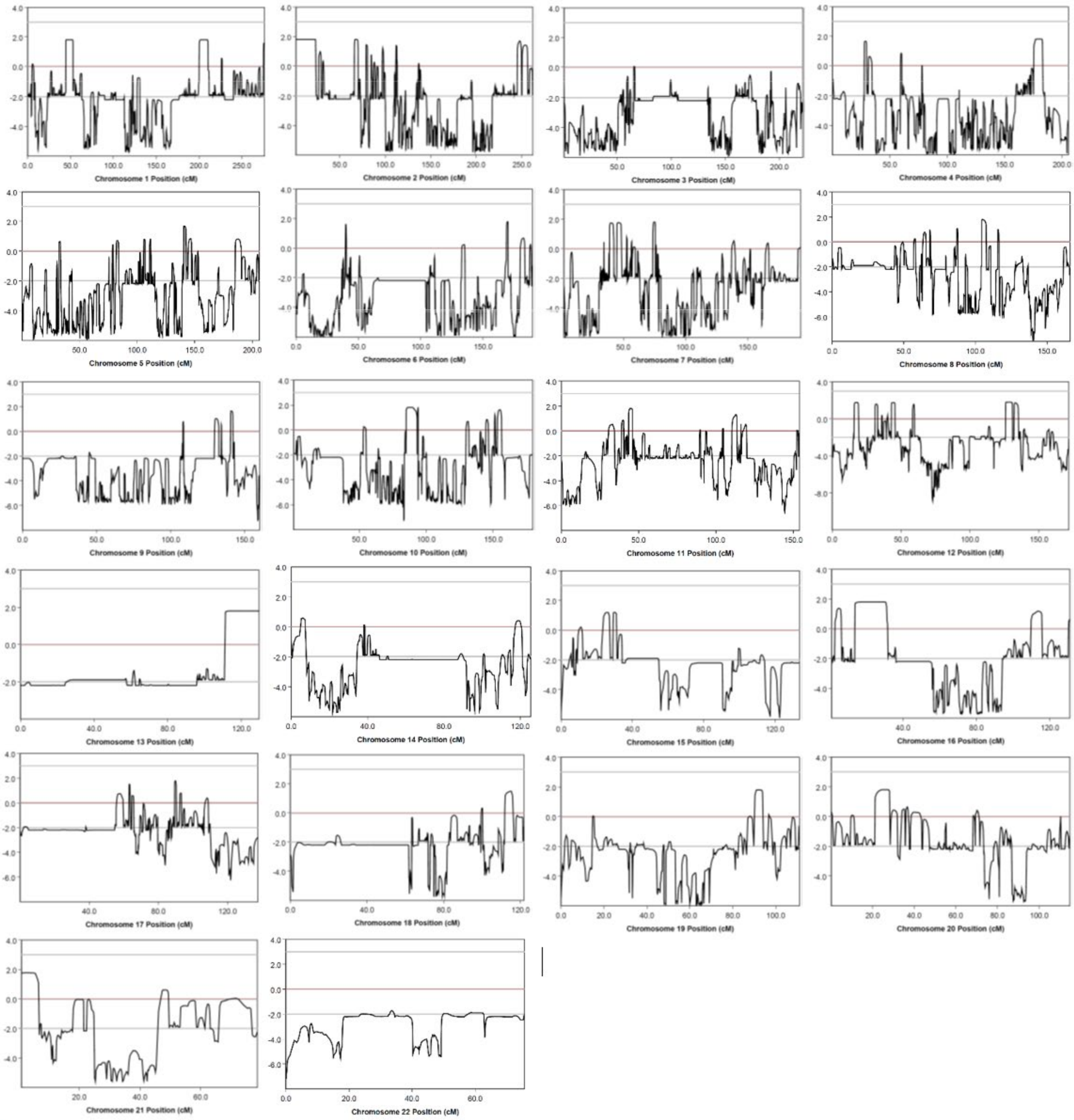

© 2024 Aloui C et al. *JAMA Network Open.*

eFigure 4: Identification of an insertion within the 3'UTR of *COL4A1* using IGV. A) Unzoomed visaualisation showing chimeric reads (2 or more colors in the same read) and reads without mate mapped (different colored reads). B) Zoom on the TSD region enabling soft-clipped reads showing the 5' and 3' sequences of the insertion. C) Cartoon to help explaining the B. TSD = Target Site Duplication (AAAAATTAAGCAA on the forward strand).

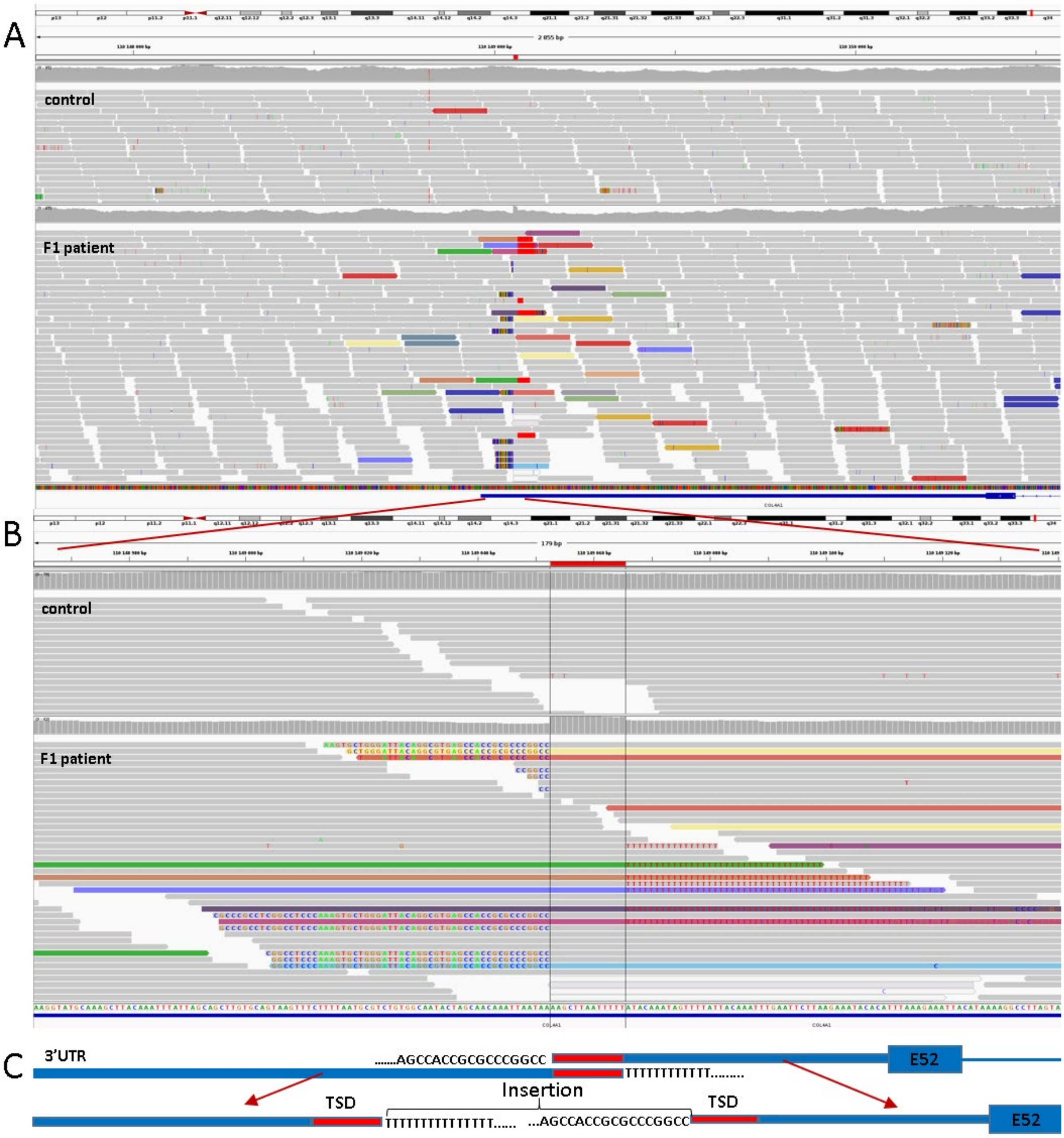

© 2024 Aloui C et al. *JAMA Network Open.*

eFigure 5: Identification of the insertion as being an AluYa5 insertion. The MEI aligned to the AluYa5 class with the exception of two variations compared to the consensus sequence (yellow rectancles). Supporting reads (soft clipped and reads without mapped mate) were extracted from the whole genome bam file of patient F1 and aligned to the consensus Alu sequences using MAFFT online software. TSD = Target Site Duplication. Black rectangles point the nucleotide different from the AluY consensus sequence. Green rectangles point to the nucleotides which differ from the AluYa8 consensus sequence. Yellow rectangles point to the nucleotides which differ from the AluYa5 consensus sequence.

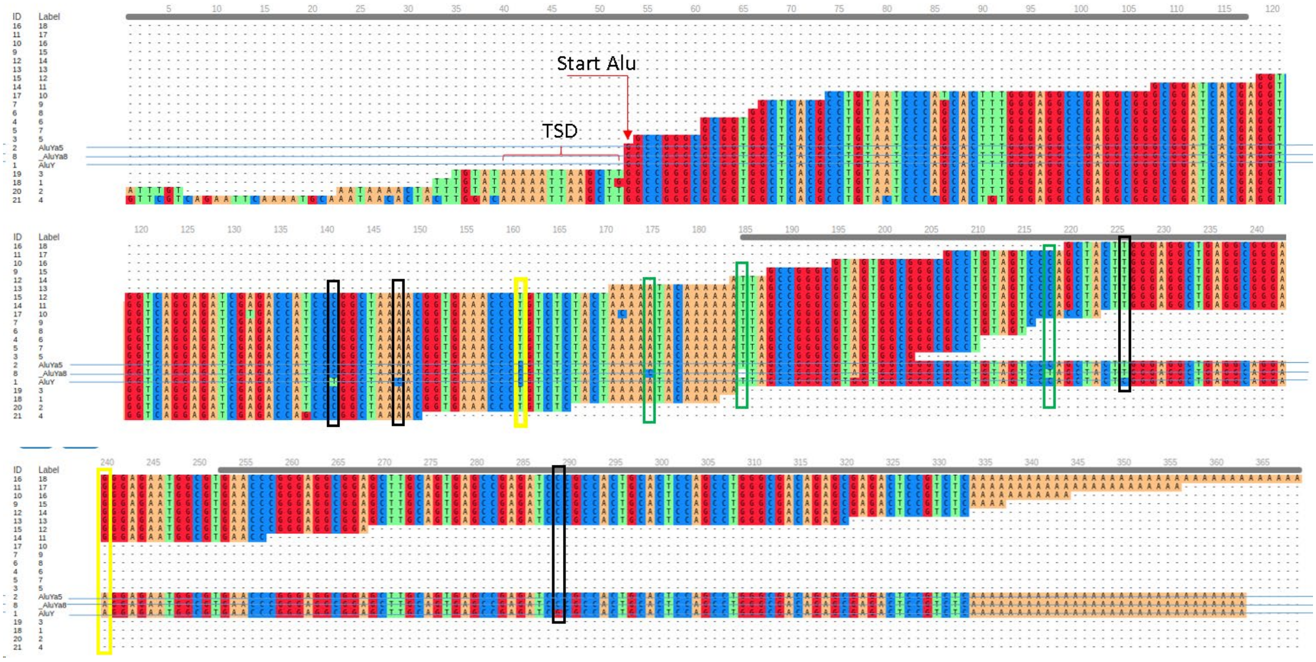

© 2024 Aloui C et al. *JAMA Network Open.*

eFigure 6: Long-reads from RNA-sequencing showing the AluYa5 insertion in the 3'UTR of COL4A1. A) A representative single long-read from nanopore RNA-seq of patient F1 (4746 nucleotides, reverse sequence). Color legend: Red = AluYa5; Purple = *COL4A1* 3'UTR; Green = TSD (AAGCTTAATTTT); Highlighted yellow: stop codon of *COL4A1*; Black: nanopore adapter.

B) DFAM database annotation identifies the insertion as an AluYa5 with two substitutions: C>T and A>G at positions 110 and 189 (blue rectangles), from the beginning of the AluYa5 consensus sequence. Model: AluYa5 consensus sequence; Query: our insertion; PP: posterior probability, or degree of confidence in each aligned residue.

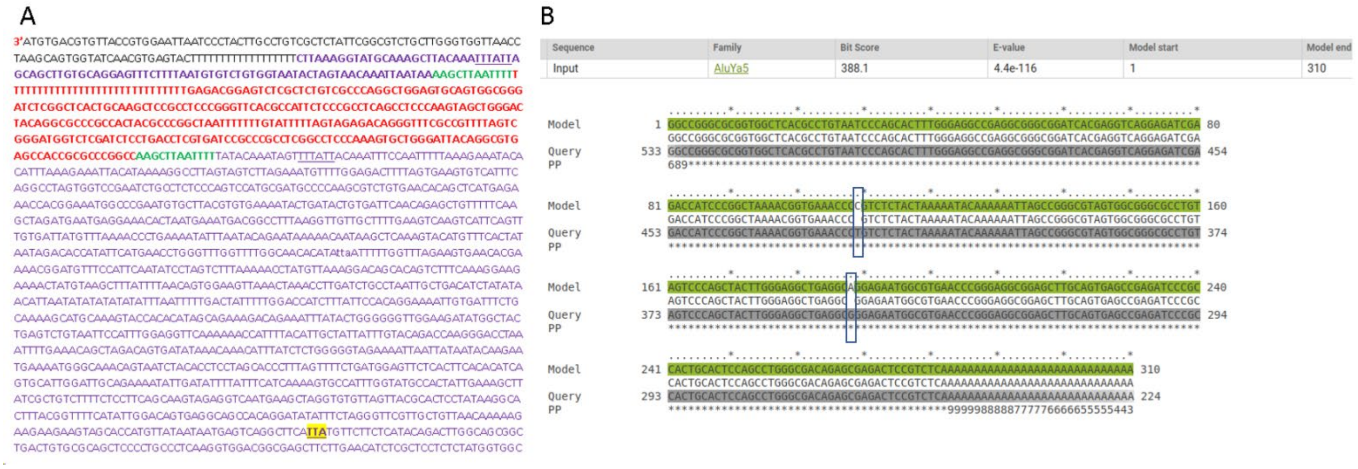

© 2024 Aloui C et al. *JAMA Network Open.*

eFigure 7: RT-qPCR analysis of wild-type and mutated *COL4A1* expressed in endogenous fibroblasts. A-B: RT-qPCR comparing mRNAs from 2 patients to 4 healthy controls and 1 CSVD control using *TBP* and *G6PD* genes for internal normalization.

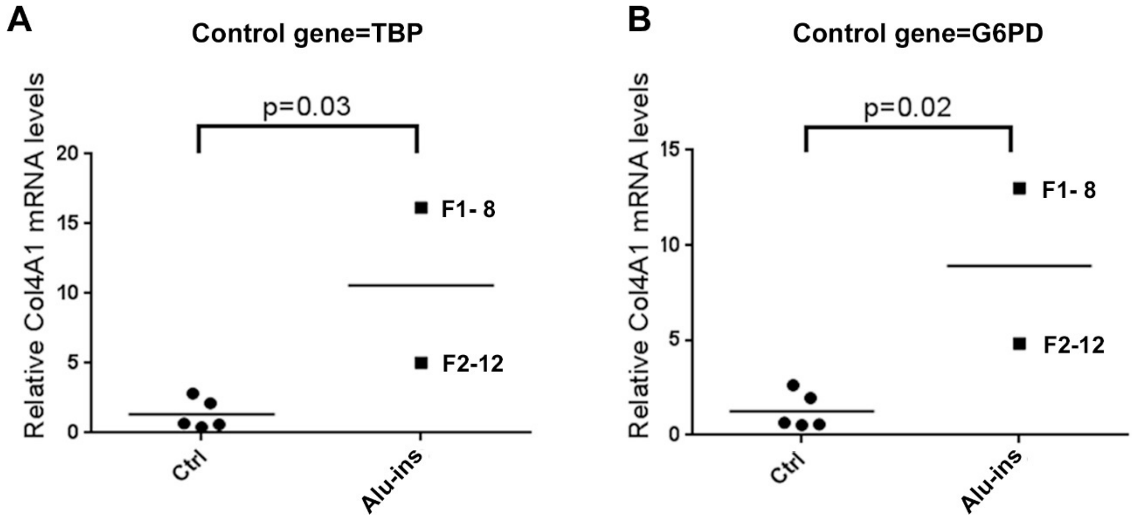

© 2024 Aloui C et al. *JAMA Network Open.*

eFigure 8: Polyadenylation signals (PAS) usage based on long-read RNAseq data analysis with IGV. A) reads from patient F1-8 showing six expressed isoforms, depending on the PAS utilized. The red color pinpoint the polyA tails. B) reads from a control individual showing mostly 2 expressed isoforms which use the 2 distal PAS. C) positions of the 6 PAS.

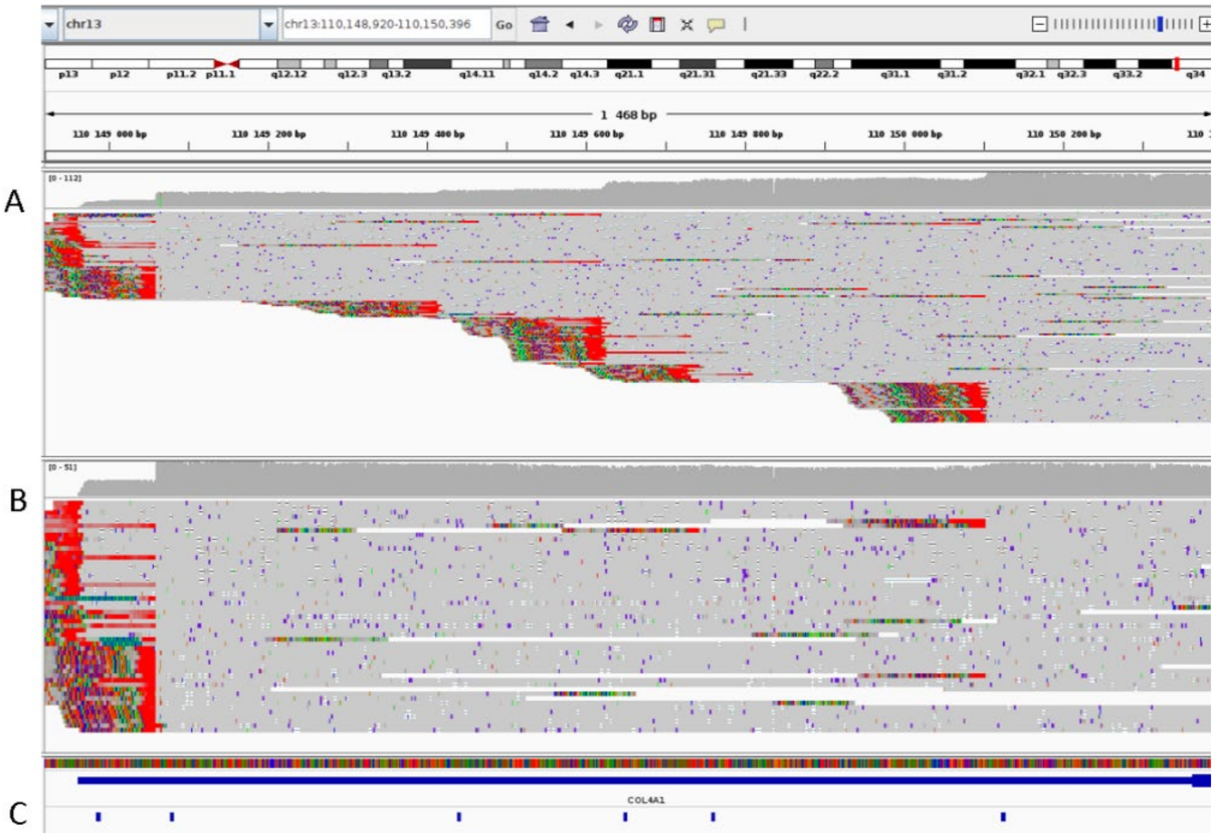

© 2024 Aloui C et al. *JAMA Network Open.*

eFigure 9: Magnetic resonance imaging data of probands from families F3-F7. Column A: Axial T1-Weighted images, B-D: Fluid-attenuated inversion recovery images. All patients present pontine infarcts and a vascular leukoencephalopathy, associated with hemispheric lacunes.

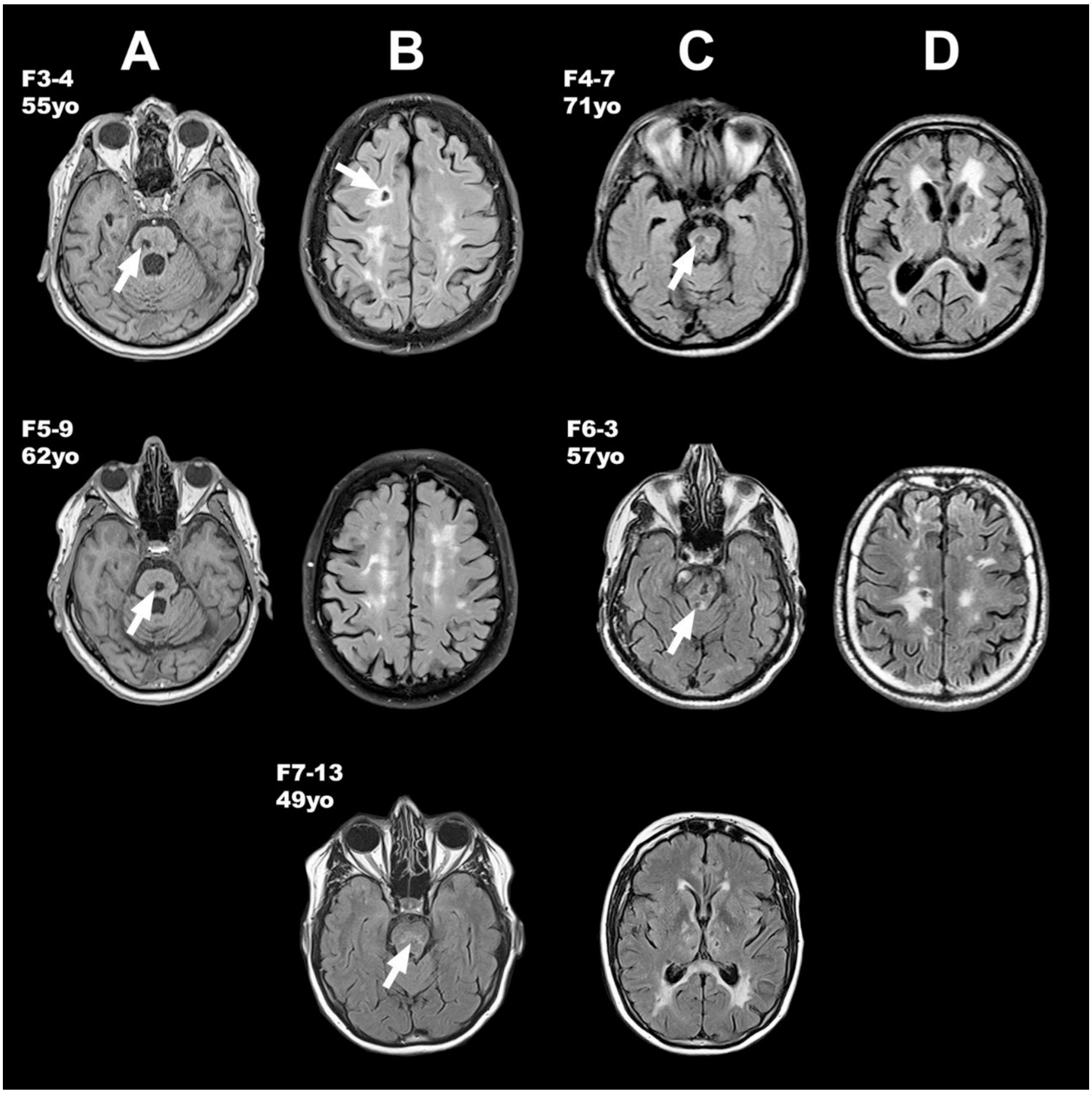

© 2024 Aloui C et al. *JAMA Network Open.*

eTable 1. Clinical features of family F1 patients.

	**F1-8**	**F1-7**	**F1-9**
Sex	M	M	F
Age at onset (years)	39	42	35
Age at time of study (yo)	39	43	41
Nb of affected relatives[1]	5	5	5
Hypertension	No	No	Yes
Symptoms at disease onset (or symptoms that led to MRI)	Sudden dizziness<br>Left arm paresthesia<br>Left central facial palsy	Sudden dizziness<br>Dysphonia	Sudden hemisensory loss
TIA history	Yes	No	No
Overt clinical isch. stroke	Yes	Yes	Yes
Cognitive impairment	Yes	Yes	No
Gait disturb.	Yes	Yes	No
Age at MRI	39	44	43
WMH	Yes	Yes	Yes
Early confluent	Yes	No	No
Confluent	No	Yes	No
Lacunes	Yes	Yes	Yes
Microbleeds	No T2*	No T2*	No

[1] Number of affected relatives based on clinical and MRI data or familial history

© 2024 Aloui C et al. *JAMA Network Open.*

eTable 2. Clinical features of family F1 patients.

	**F2-9**	**F2-10**	**F2-11**	**F2-12**	**F2-13**	**F2-16**
Sex	M	M	M	F	M	F
Age at onset (years)	50	42	54	59	59	60
Age at time of study (yo)	53	42	55	66	59	60
Nb of affected relatives[1]	8	8	8	8	8	8
Hypertension	Yes	Yes	No	Yes	No	No
Symptoms at disease onset (or symptoms that led to MRI	Chronic headache<br>Cognitive impairment	Transient diplopia	Diplopia<br>Right eye ptosis	Left hemiparesia	Left III nerve paresis	Numbness of the left hemibody<br>Gait disturbance
TIA history	No	Yes	No	No	No	No
Overt clinical Isch. stroke	No	Yes	Yes	Yes	Yes	Yes
Cognitive impairment	Yes	No	Yes	No	No	No
Gait disturb.	No	Yes	Yes	No	No	No
Age at MRI	53	55	54	60	59	60
WMH	Yes	Yes	Yes	Yes	Yes	Yes
Early confluent	-	-	Yes	No	Yes	No
Confluent	Yes	Yes	No	No	No	No
Lacunes	Yes	Yes	Yes	No	Yes	Yes
Microbleeds	No	Yes	No	No	No	No

[1] Number of affected relatives based on clinical and MRI data or familial history

© 2024 Aloui C et al. *JAMA Network Open.*

eTable 3. Clinical features of families F3-F7 probands.

	**F3-4**	**F4-13**	**F5-9**	**F6-3**	**F7-13**
Sex	M	M	M	M	M
Age at onset (yo)	48	55	55	38	40
Age at time of study (yo)	55	55	55	57	49
No. of affected relatives	2	2	2	1	8
Hypertension	Yes	No	No	No	No
Symptoms at disease onset (or symptoms that led to MRI)	Internuclear ophtalmoplegia	Hemiplegia	Dysarthria<br>Cerebellar ataxia	Right hemibody motor sensory deficit	Gait disturbance
History of TIA	No	No	No	No	No
Ischemic stroke	Yes	Yes	Yes	Yes	Yes
Cognitive impairment	No	No	Yes	Yes	Yes
Gait disturbance	Yes	Yes	Yes	Yes	Yes
Age at MRI (years)	55	55	62	57	49
WMH	Yes	Yes	Yes	Yes	Yes
Early confluent	Yes	Yes	No	No	No
Confluent	No	No	Yes	Yes	No
Lacunes	Yes	Yes	Yes	Yes	Yes
Microbleeds	Yes	Yes	Yes	Yes	Yes

© 2024 Aloui C et al. *JAMA Network Open.*

eTable 4. Clinical features of families F3-F7 affected relatives.

	**F3-2**	**F3-5**	**F4-7**	**F5-5**	**F5-10**	**F7-11**	**F7-14**
Sex	F	M	M	F	F	F	F
Age at onset (years)	69	52	39	60	61	46	45
Age at time of study (years)	78	54	70	Deceased at 70 yo	61	57	45
No. of affected relatives[1]	2	2	2	2	2	8	8
Hypertension	Yes	No	No	Unknown	No	Yes	No
Symptoms at disease onset (or symptoms that leed to MRI)	Pontine infarct	Right hemibody paresthesia	Uncl ear	Dizziness Vomiting<br>Diplopia	Gait disturbance left tinnitus	Partial IIIrd nerve paresis<br>Dysarthria<br>Ataxia	Left bracchiofacial paresthesia<br>Right internuclear ophtalmoplegia<br>Diplopia<br>Gait disturbance
History of TIA	No	Yes	No	No	No	Yes	No
Ischemic stroke	Yes	Yes	Yes	Yes	Yes	Yes	Yes
Cognitive impairment	Yes	No	Yes	Yes	No	Yes	No
Dementia	Yes	No	Yes	No	No	Yes	No
Gait disturbance	Yes	No	Yes	Yes	Yes	Yes	Yes
Age at MRI (years)	76	52	71	60	61	57	45
WMH	Yes	Yes	Yes	Yes	Yes	Yes	Yes
Early confluent	No	No	Yes	Yes	Yes	No	No
Confluent	No	No	No	No	No	Yes	No
Lacunes	Yes	Yes	Yes	Yes	Yes	Yes	Yes
Microbleeds	No	Yes	No T2*	Yes	Yes	Yes	No

[1] Number of affected relatives based on clinical and MRI data or familial history

© 2024 Aloui C et al. *JAMA Network Open.*

eMethods: Detailed Methods

Linkage Analysis

Linkage analysis for Families F1 and F2 was performed with data obtained from Illumina HumanOmniExpress-24v1-0 array, ~712000 SNPs (Illumina, San Diego, California, USA). Genotyping was done as previously described.[1] Genotypes were extracted with GenomeStudio v2 software, (Illumina). Data were, then processed with Alohomora v0.33 software[2] and whole genome parametric linkage analysis were done with Merlin software[3] with the following parameters: one marker every 100 kb with MAF > 0.05 in Caucasians of HapMap project, a dominant model, full penetrance, no phenocopy and a disease allele frequency of 0.0001. Three and five affected members were analyzed for F1 and F2, respectively (Fig. 1). Intervals reaching the maximum theoretical logarithm of odds score (Lod Score) achievable in these two families (0.9 and 1.8, for F1 and F2, respectively) were considered as possibly linked.

Exome sequencing

Whole exome sequencing of 244 CSVD patients, collected between 2018 and 2020, as well as that of individuals from F1 and F2 families was performed on the Integragen platform (Integragen, Evry, France) using the SureSelectXT Human All Exon V5+UTR kit (Agilent Technologies, Santa Clara, CA). Pair-end reads were mapped to the hg38 human genome using BWA-MEM and variant calling was performed with HaplotypeCaller according to GATK best practices.[4–6] Variant annotation for functional consequences and allele frequencies was performed with VEP v95.[7]

To be considered as a candidate variant, high impact (i.e. stop gain, frameshift, canonical splice sites and start/stop lost) and missense variants (predicted pathogenic with PolyPhen2) should i) be rare (frequency < 1% in an in-house dataset of 1200 exomes sequenced in the same platform as our exomes, frequency < 0.01% in any public databases: ESP6500, 1000 Genomes, gnomAD v2.1 and Topmed freeze 5), ii) cosegregate with the affected phenotype in at least one of the two families F1-2, and iii) the gene carrying this variant should be mutated in at least an additional proband in the WES cohort. If so, we performed a Fisher exact collapsing test using gnomAD as controls as in Aloui et al.[8]

© 2024 Aloui C et al. *JAMA Network Open.*

Whole genome sequencing

To further investigate the structural and regulatory non-coding variant content in the linked locus, we performed PCR-free whole genome sequencing (WGS) of the three patients of family F1. WGS was carried out by Integragen (Integragen, Evry, France) with paired-end 150bp reads on an Illumina NovaSeq machine. Read alignment and short variant calling was same as for the WES data.

Structural variation screening

Copy number variation (large duplication and deletions) were searched from Illumina SNP array data as previously described[1]. Structural variations from WGS data were searched by Delly[10], Lumpy[11], and Manta[12] tools and annotated by AnnotSV[13]. Soft clipped reads were further visually inspected using IGV v2.9[14]. The Mobile Element Locator Tool (MELT)[15] was then used to discover and genotype mobile element insertions (MEI), namely SINEs, LINEs, SVA, and HERVK in the sequenced three WGS and also in all the available WES data.

COL4A1/COL4A2 mRNA quantification

RNA was extracted and quantified from cultured patients' and controls' dermal fibroblasts as previously described.[9] For each sample, 1 µg of RNA was reverse-transcribed according to the manufacturer's instructions (M-MLV Reverse Transcriptase; Life Technologies, Carlsbad, CA). Reverse transcription and quantitative polymerase chain reaction (RT-qPCR) was performed in a LightCycler480 system (Roche, Basel, Switzerland) using iQ SYBR Green Supermix (Bio-Rad, Hercules, CA). All reactions were performed in triplicate. mRNA levels were normalized to *TBP* and *G6PD* mRNA levels using the $2^{\Delta\Delta Ct}$ method.

COL4A1 protein level quantification

Proteins from patients and controls' cultured fibroblasts were treated with RIPA buffer (Boston BioProducts, USA) and protease inhibitor (Thermo Scientific, USA). Five µg of proteins were resolved by polyacrylamide gel electrophoresis and transferred to PVDF membrane. The blots were blocked with 1% TBS1X-T0 and 5% milk and incubated overnight at 4°C with primary antibodies followed by incubation with HRP conjugated secondary antibodies. Goat Anti-Type IV Collagen-AF488 antibody,

diluted 1:1000 (Cat. No.: 1340-30, SouthernBiotech) was used for collagen IV quantification with anti-Goat-HRP diluted 1:10 000. Lysates were normalized using an antibody against Fibronectin diluted 1:1000 (AF1918, R&D Systems) with a Rabbit anti-sheep-HRP diluted 1:3000 (ab6747, Abcam). Proteins from conditioned medium were normalized with rabbit-anti-MMP2 diluted 1:2000 (ab92536, Abcam) with anti-rabbit-HRP diluted 1:3000 (ref 7076, Cell Signaling Technology). Detection was carried out using the c300 imaging system, and AzurSpot 2.0 analysis software (Azure Biosystems).

PCR and Sanger sequencing

The identified MEI in the 3'UTR of *COL4A1* was validated by Sanger sequencing and genotyped by standard PCR. Specific primers were designed with Primer3 to amplify the insertion [16]. The used PCR primers were: COL4A1 forward 5'-CCATTTCCGTGGTTTCTCAT-3' and reverse 5'-AGGCGACGAAAGAGGAAGA-3'; with a classical PCR procedure and the amplicons were revealed with 2% agarose electrophoresis gel (protocols and results available on request). The PCR products were sequenced with forward and reverse primers using an ABI 3730 capillary sequencer (Applied Biosystems, Life Technologies).

In addition, PCR was performed to genotype the MEI in all the patients and in the 467 healthy French controls.

Long read RNA sequencing

Total RNA was extracted from cultured fibroblasts of three patients (F1-8, F2-12 and F8-16), 4 healthy controls and 3 CSVD patients that did not carry the MEI, using the TRIzol reagent (Thermo Fisher), following the manufacturer's protocols. RNA integrity was evaluated by the Agilent 2100 Bioanalyzer (Agilent Technologies).

Library preparation and Nanopore sequencing were performed at the Ecole Normale Superieure genomics core facility (Paris, France). Ten ng of total RNA were amplified and converted to cDNA using SMART-Seq v4 Ultra Low Input RNA kit (Clontech). Afterwards an average of 13 fmol of amplified cDNA was used to prepare library following SQK-PBK004 kit (PCR Barcoding kit; ONT). After the PCR adapter ligation, a 0,6X Agencourt Ampure XP beads clean-up was optimized and 2 fmol

© 2024 Aloui C et al. *JAMA Network Open.*

of the purified product was taken into PCR for amplification and barcodes addition with a 17 minutes elongation at each 18 cycles.

Samples were pooled in equimolar quantities to obtain 30 fmol of cDNA and the rapid adapter ligation step was performed. Libraries were multiplexed by 4 on 3 R9.4.1 flowcells according to the manufacturer's protocol. The two patients F1-8 and F2-12 were sequenced in triplicates, one replicate in each run. Sequencing was performed with the SQK-PBK004 72-hour sequencing protocol run on the MinION MkIC, using the MinKNOW software (versions 21.11.7). A mean of 2,7 ± 1,5 million passing ONT quality filter reads was obtained for each of the10 samples. Base-calling from read event data was performed by Guppy (version 6.0.1).

The analyses were performed using the Eoulsan pipeline[17], including read filtering, mapping and alignment filtering: Before mapping, poly N read tails were trimmed and discarded. Reads were then aligned against the human genome (GRCh38.p13) from Ensembl version 105 using minimap2 (version 2.17)[18] with arguments -x splice --secondary=no --junc-bed using Homo sapiens Ensembl v105 bedfile.

Alternative Polyadenylation signal usage

To quantify the various transcribed isoforms depending on the presence or absence of the MEI in the 3'UTR of *COL4A1*, we used the BAM files visualized in IGV and we manually counted the reads that contained poly(T) stretches on their 3'ends, stretches that are preceded by a PAS listed in the PolyA_DB3 database.[19] Six PAS have been identified with at least one read in the patients and controls. The relative usage of each PAS was analyzed by the ratio between the number of reads in which this given PAS was immediatly preceding the PolyT tail and the total number of reads carrying a polyT tail.

Statistical analyses

The number of carriers of the candidate AluYa5 insertion was compared between cases and controls using Fisher's exact test implemented in R v4.2 (https://www.r-project.org). mRNA and protein levels between cases and controls were compared using the Mann-Whitney test using GraphPad Prism v9 software. Differential use of polyadenylation sites between patients and controls was analyzed with a Mann-Wittney test using R v4.2. Statistical significance threshold was set to 0.05.

© 2024 Aloui C et al. *JAMA Network Open.*

eReferences

1. Aloui C, Guey S, Pipiras E, et al. Xq28 copy number gain causing moyamoya disease and a novel moyamoya syndrome. *Journal of Medical Genetics*. 2020;57(5):339-346. doi:10.1136/jmedgenet-2019-106525

2. Rüschendorf F, Nürnberg P. ALOHOMORA: a tool for linkage analysis using 10K SNP array data. *Bioinformatics*. 2005;21(9):2123-2125. doi:10.1093/bioinformatics/bti264

3. Abecasis GR, Cherny SS, Cookson WO, Cardon LR. Merlin—rapid analysis of dense genetic maps using sparse gene flow trees. *Nature Genetics*. 2002;30(1):97-101. doi:10.1038/ng786

4. DePristo MA, Banks E, Poplin R, et al. A framework for variation discovery and genotyping using next-generation DNA sequencing data. *Nature Genetics*. 2011;43(5):491-498. doi:10.1038/ng.806

5. Van der Auwera GA, Carneiro MO, Hartl C, et al. From FastQ data to high confidence variant calls: the Genome Analysis Toolkit best practices pipeline. *Curr Protoc Bioinformatics*. 2013;43:11.10.1-11.10.33. doi:10.1002/0471250953.bi1110s43

6. Picard Toolkit repository. Picard Toolkit repository. Published 2019. Accessed May 4, 2020. http://broadinstitute.github.io/picard/

7. McLaren W, Gil L, Hunt SE, et al. The Ensembl Variant Effect Predictor. *Genome Biology*. 2016;17(1):122. doi:10.1186/s13059-016-0974-4

8. Aloui C, Hervé D, Marenne G, et al. End-Truncated LAMB1 Causes a Hippocampal Memory Defect and a Leukoencephalopathy. *Ann Neurol*. Published online October 20, 2021:ana.26242. doi:10.1002/ana.26242

9. Verdura E, Hervé D, Bergametti F, et al. Disruption of a miR-29 binding site leading to COL4A1 upregulation causes pontine autosomal dominant microangiopathy with leukoencephalopathy. *Annals of Neurology*. 2016;80(5):741-753. doi:https://doi.org/10.1002/ana.24782

10. Rausch T, Zichner T, Schlattl A, Stütz AM, Benes V, Korbel JO. DELLY: structural variant discovery by integrated paired-end and split-read analysis. *Bioinformatics*. 2012;28(18):i333-i339. doi:10.1093/bioinformatics/bts378

11. Layer RM, Chiang C, Quinlan AR, Hall IM. LUMPY: a probabilistic framework for structural variant discovery. *Genome Biol*. 2014;15(6):R84. doi:10.1186/gb-2014-15-6-r84

12. Chen X, Schulz-Trieglaff O, Shaw R, et al. Manta: rapid detection of structural variants and indels for germline and cancer sequencing applications. *Bioinformatics*. 2016;32(8):1220-1222. doi:10.1093/bioinformatics/btv710

13. Geoffroy V, Herenger Y, Kress A, et al. AnnotSV: an integrated tool for structural variations annotation. *Bioinformatics*. 2018;34(20):3572-3574. doi:10.1093/bioinformatics/bty304

14. Robinson JT, Thorvaldsdóttir H, Winckler W, et al. Integrative Genomics Viewer. *Nat Biotechnol*. 2011;29(1):24-26. doi:10.1038/nbt.1754

15. Gardner EJ, Lam VK, Harris DN, et al. The Mobile Element Locator Tool (MELT): population-scale mobile element discovery and biology. *Genome Res*. 2017;27(11):1916-1929. doi:10.1101/gr.218032.116

© 2024 Aloui C et al. *JAMA Network Open.*

16. Untergasser A, Cutcutache I, Koressaar T, et al. Primer3--new capabilities and interfaces. *Nucleic Acids Res*. 2012;40(15):e115. doi:10.1093/nar/gks596

17. Jourdren L, Bernard M, Dillies MA, Le Crom S. Eoulsan: a cloud computing-based framework facilitating high throughput sequencing analyses. *Bioinformatics*. 2012;28(11):1542-1543. doi:10.1093/bioinformatics/bts165

18. Li H. New strategies to improve minimap2 alignment accuracy. *Bioinformatics*. 2021;37(23):4572-4574. doi:10.1093/bioinformatics/btab705

19. Wang R, Nambiar R, Zheng D, Tian B. PolyA_DB 3 catalogs cleavage and polyadenylation sites identified by deep sequencing in multiple genomes. *Nucleic Acids Research*. 2018;46(D1):D315-D319. doi:10.1093/nar/gkx1000

© 2024 Aloui C et al. *JAMA Network Open.*
