# Supplementary material for: An AluYa5 Insertion in the 3′UTR of COL4A1 and Cerebral Small Vessel Disease
Source: JAMA Netw Open. 2024 Apr 17;7(4):e247034. doi: 10.1001/jamanetworkopen.2024.7034 (PMC11024774; doi:10.1001/jamanetworkopen.2024.7034)
Supplement: Supplement 2. — Data Sharing Statement [file jamanetwopen-e247034-s002.pdf]

Data Sharing Statement

Aloui. An AluYa5 Insertion in the 3′UTR of *COL4A1* and Cerebral Small Vessel Disease. *JAMA Netw Open*. Published online April 17, 2024. doi:10.1001/jamanetworkopen.2024.7034

Data
Data available: No

Additional Information
Explanation for why data not available: Data sharing of whole exome and whole genome sequencing patients' is not possible as according with the confidentiality rules implemented for genetic data in France.
